# Extraction and Application of Natural Rutin From *Sophora japonica* to Prepare the Novel Fluorescent Sensor for Detection of Copper Ions

**DOI:** 10.3389/fbioe.2021.642138

**Published:** 2021-02-22

**Authors:** Shilong Yang, Lu Sun, Zhiwen Song, Li Xu

**Affiliations:** ^1^Advanced Analysis and Testing Center, Nanjing Forestry University, Nanjing, China; ^2^College of Science, Nanjing Forestry University, Nanjing, China

**Keywords:** rutin, (2-hydroxypropyl)-β-cyclodextrin, fluorescent sensor, copper ions, *Sophora japonica*

## Abstract

Rutin (**R**), a representative flavonoid found in various biomasses, can be used to prepare different fluorescent sensors for environmental, biological and medical fields. In this work, the natural **R** in *Sophora japonica* was extracted and purified to prepare fluorescent-responding sensor systems intended to recognize copper ions with both strong selectivity as well as appropriate sensitivity. Results showed that neat **R** had no obvious fluorescent emission peak in PBS buffer solution. However, when **R** and (2-hydroxypropyl)-β-cyclodextrin (**CD**) were introduced within buffer solution, fluorescent emission intensity was significantly increased due to the resultant **R-CD** inclusion complex. In addition, the formed **R-CD** inclusion complex was shown to behave as the aforementioned fluorescent sensor for copper ions through a mechanism of quenched fluorescent emission intensity when **R-CD** became bound with copper ions. The binding constant value for **R-CD** with copper ions was 1.33 × 10^6^, allowing for quantification of copper ions between the concentration range of 1.0 × 10^–7^–4.2 × 10^–6^mol⋅L^–1^. Furthermore, the minimum detection limit was found to be 3.5 × 10^–8^mol⋅L^–1^. This work showed the prepared **R-CD** inclusion complex was both highly selective and strongly sensitive toward copper ions, indicating that this system could be applied into various fields where copper ions are of concern.

## Introduction

As the shortage of oil resources and environmental pollution become more serious, biomass resources have attracted lots of attention as sustainable alternatives. Transformation of the constituents (cellulose, hemicellulose, lignin) into value-added products such as reinforcing materials, food additives, adhesive, and more continue to be extensively investigated (Du et al., 2019; Sun et al., 2019; Zhou and Xu, 2019; Zhou et al., 2019; Huang et al., 2020; Pei et al., 2020b; Zhao et al., 2020). Apart from the structural constituents of biomass, there is also potential value in the variously minor constituents present, specifically the flavonoids and polyphenols. The polyphenols can showed different physical and biological activity in bio-materials (Dong et al., 2020; Pei et al., 2020a; Zheng et al., 2021). Flavonoids are widely present in most plants. Biological activities of flavonoids are reported and reviewed frequently in the literature (Singh et al., 2014; Zhao et al., 2016). Nevertheless, there are few reports about fluorescence properties of flavonoids. In one relevant work, the authors found that flavonoids isolated from bamboo residues were capable of detecting Fe^3+^ *in vitro* (Su et al., 2019). In a different work, the flavonoid quercetin was used to provide bioimaging in two applicable biological mediums (He et al., 2018). The key element of both of these works is that they demonstrate that flavonoids could play an important role as fluorescent chemicals from sustainable resources.

**Scheme 1:**
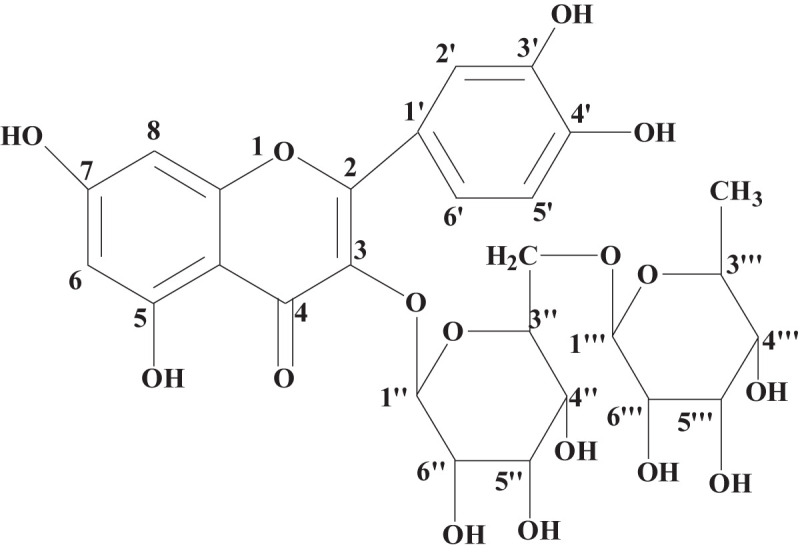
Structure of the rutin.

Rutin (**R,** Scheme 1) is a representative flavonoid that can be found in various plants. It has displayed many favorable biological activities, including anti-inflammatory, antiviral, and others (Nkpaa et al., 2019; Huynh et al., 2020; Wani et al., 2021). However, there are few works which have sought to isolate **R** from biomass and use it as the fluorescent sensor for detection of metal ions. Therefore, this was the task of the present document. Thousands of ion-detective fluorescent sensors for ion have been reported upon in the literature (Zhang et al., 2018; Chen et al., 2019; Kan et al., 2020; Li et al., 2021). Importantly, the most common theme in these studies is that the fluorescent molecules investigated were often formed synthetically and their workup often included use of toxic reagents and solvents. Compared to these more traditional fluorescent sensors, **R** is easily extracted from plants with neat water or aqueous ethanol solutions. In addition, **R** is a dietary supplement and has excellent biocompatibility.

*Sophora japonica* is dried flower bud of *Sophora japonica* trees, which are planted in China widely for its ecological and economic benefits. However, the flowers of *Sophora japonica* trees fall and become waste. There is lots amount of **R** in the flowers, especially in the flower buds. To make full use of *Sophora japonica*, **R** is extracted from *Sophora japonica* and purified to prepare the novel fluorescent sensor for detection of different metal ions in this study. Isolated **R** was complexed with 2-hydroxypropyl-β- cyclodextrin (**CD**) in a buffer solution (pH = 7.40) to improve its emission fluorescence, and this resulting complex (**R-CD)** was then applied for detection and quantitation of metal ions. The mechanism of changes to fluorescence was also discussed to understand the ability of **R**-based fluorescent sensor. It is hoped that this work will shine light upon a new approach toward sensor molecules from the approach of utilization of secondary plant substance.

## Materials and Methods

### Reagents and Instruments

The different metal salts (AR) were provided by the Nanjing Reagent Co., Ltd. Methyl alcohol (HPLC) was produced by Tedia Company, Inc. **R** was extracted from *Sophora japonica* acquired from Bozhou Good health Food Co., Ltd. 2-Hydroxypropyl-β-cyclodextrin was purchased from Aladdin Reagent Co., Ltd. Next, phosphate buffer solution (PBS, pH = 7.40) were obtained from Beijing Solarbio Science & Technology Co., Ltd. Finally, sodium tetraborate, hydrochloric acid and calcium hydroxide were purchased by Sinopharm Chemical Reagent Co., Ltd. It is important to note that all solutions involving the above mentioned reagents were prepared freshly using ultrapure water produced by Milli-Q.

Fluorescence spectra and UV-visible absorption spectra were recorded on a PerkinElmer LS55 spectrophotometer and a Lambda 950 spectrophotometer, respectively. All ^1^H NMR spectra were obtained via a Bruker AVANCE III HD 600 MHz spectrometer. Finally, FT-IR spectra were obtained by way of a Bruker VERTEX 80V.

### Extraction and Purification of **R**

To begin extraction, dried *Sophora japonica* was added into a boiling aqueous solution of 0.4% sodium tetraborate. To reach a pH between 8 and 9, calcium hydroxide was added into the solution. After 0.5 h, the solution was filtered with gauze and filter paper, respectively. We observed that the temperature of the solution needed to remain above 60°C in order to avoid unwanted precipitation of **R**. Next, an appropriate amount of hydrochloric acid solution was added to the filtrate to render the solution at a pH between 2 and 3. After this adjustment, a mass of crude **R** precipitated from solution. Crude **R** was then re-dissolved in boiling water, and the hot solution was then quickly filtered again. From this filtrate, the **R** recrystallized while it cooled. The crystals (purified **R**) were then collected via vacuum filtration and allowed to dry.

The ^1^H NMR spectra of purified **R** was shown in Supplementary Figure 1, and the data was as follows: ^1^H NMR (DMSO-d_6_ 600 MHz): 12.594 (1H, s, 5-OH), 10.833 (1H, s,7-OH), 9.670 (1H, s, 3′-OH), 9.176 (1H, s, 4′-OH), 7.548 (2H, m, 2′-H, 6′-H), 6.842 (1H, d, J = 8.16 Hz, 5′-H), 6.386 (1H, d, J = 2.04 Hz, 8-H), 6.195 (1H, d, J = 2.04 Hz, 6-H), 5.344 (1H, d, J = 7.32 Hz, 1′′-H), 4.385 (1H, s, 1′′′-H), 4.346–4.384 and 4.527–5.283 (6H, m, surgar moieties-OH), 3.058–3.714 (10H, m, surgar moieties-H), 0.991 (3H, d, J = 6.18 Hz, surgar moieties-CH_3_). The results confirmed **R** was successfully extracted from *Sophora japonica* (Nam et al., 2015; Yingyuen et al., 2020).

### Preparation of the Stock Solutions

Stock solutions (1 × 10^–2^ mol⋅L^–1^) of different metal salts were prepared by dissolving appropriate amount of metal salts into ultrapure water. The stock solutions of **R** and **CD** were prepared by dissolving the appropriate amounts of **R** and **CD** into methyl alcohol and PBS solutions, respectively. The **R-CD** solution was prepared by adding the **R** solution into the **CD** solution at a volumetric ratio 1:99. The concentration of **R** in **R-CD** solution was 1 × 10^–5^ mol⋅L^–1^, while the concentration of **CD** varied by experiment.

### Optimizing Concentration of **CD**

To study the effect of **CD** concentration on the fluorescence emission intensity, **R-CD** solutions with different concentrations of **CD** were measured via fluorescent spectrophotometry at the excitation wavelength 425 nm (Supplementary Figure 2). The concentrations corresponding to stronger fluorescence emission intensity were considered during these experiments.

### Selectivity and Sensitivity of **R-CD** on Copper Ions

In order to evaluate the sensor system’s selectivity for copper or other ions, stock solutions of different metal salts were added into individual **R-CD** solutions. And the fluorescence emission intensities were measured to study the changes after adding metal ions. For the mixed solutions, the concentration of copper ions was controlled at 1 × 10^–5^ mol⋅L^–1^, the other metal ions was controlled at 2 × 10^–5^ mol⋅L^–1^, or 5 × 10^–5^ mol⋅L^–1^.

To evaluate the influence from other metal ions on the process of detecting copper ions, a pre-determined amount of copper ions was added into **R-CD** solutions to produce **R-CD-Cu(II)** complexes. Next, the other investigated metal ions were introduced into the **R-CD-Cu(II)** complex. And the fluorescence emission intensities of **R-CD-Cu(II)** complexes were measured.

In effort to evaluate the strength of correlation between fluorescence emission intensity and copper ions concentration, fluorescence titration experiments were performed. From this, fluorescence emission intensities of **R-CD** with various concentrations of copper ions were measured.

### Stoichiometric Ratio

The stoichiometric ratio between the **R** and copper ions in **R-CD-Cu(II)** complex was determined by Job’s method according to the literature (Facchiano and Ragone, 2003).

### Detecting Copper Ions in Real Samples

The ability of **R-CD** to recognize copper ions in a real sample was verified by designing experiments to detect copper ions in various solutions prepared in our laboratory.

## Results and Discussion

### Relationship Between **CD** Concentration and Fluorescent Intensity

**FIGURE 1:**
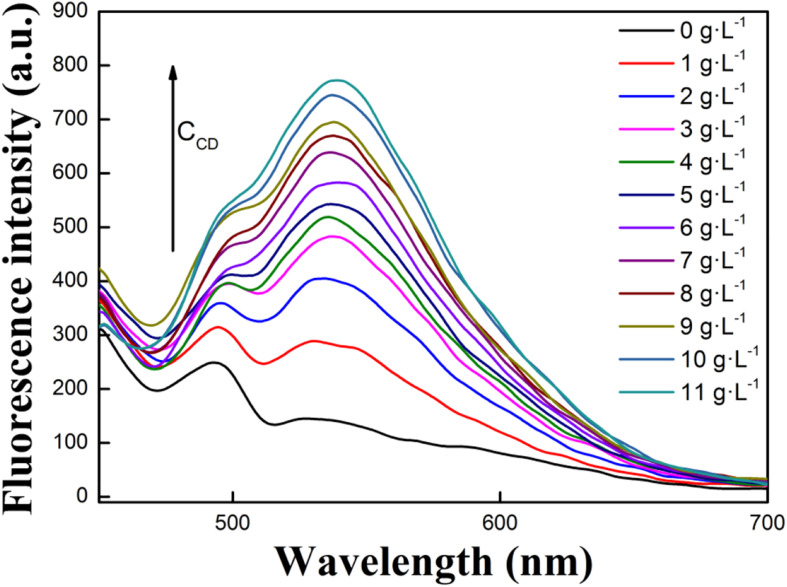
Effects of **CD** concentration on the fluorescence intensity of **R** in buffer solution (1:99, V/V, pH = 7.40, [**R**] = 1.0 × 10^– 5^ mol⋅L^– 1^).

To study how concentration of **CD** impacts fluorescence emission intensity of **R**, increasing amounts of **CD** were added to solution and respective fluorescence emission spectra of **R** were recorded (Figure 1). To begin, it is evident that **R** and **CD** had no obvious emission peaks between 510 and 700 nm. When adding **R** into buffer solution containing **CD**, the fluorescence emission peak at 535 of **R** became stronger when increasing [**CD**]. However, once the concentration of **CD** reached 10 g⋅L^–1^, no further increase to fluorescence emission intensity was observed. Thus, 10 g⋅L^–1^ was selected as an experimental condition for follow-up experiments. Overall, these spectra demonstrated that the interaction between **R** and **CD** occurs, and that the **R-CD** inclusion complex forms. To understand the interaction, FT-IR and ^1^HNMR spectra of **R**, **CD,** and **R-CD** were studied.

When observing the acquired FT-IR spectra (Supplementary Figure 3), it was found that **R-CD** possessed all peaks that both **R** and **CD** had when alone. However, the absorption band of polyhydroxyl from **R-CD** became narrower compared to **R**. This change indicates that **R** interacts with **CD** through at least one of its multiple hydroxyl functional groups. Further evidence supporting the inclusion of **R** inside the cavity of **CD** was obtained by ^1^H NMR. The ^1^H NMR spectra of **R**, **CD** and **R-CD** can be found in Supplementary Figure 4. It is seen in the spectra that some peaks of **R** in **R-CD** either weakened or disappeared, along with broadening and up-field shifts of some peaks taking place (Table 1). These results supported the hypothesis regarding inclusion of **R** inside the cavity of **CD** via interaction around hydroxyl functionalities.

**TABLE 1:** The chemical shifts of **R-CD** and **R**.

R/ppm	R-CD/ppm	R/ppm	R-CD/ppm
12.6096	12.5930	5.3660	5.3467
10.8489	–	5.3537	5.3344
9.6851	–	5.2987	5.2741
9.1910	–	5.1240	–
7.5653	7.5446	5.0938	–
7.5617	7.5306	4.5427	–
7.5484	7.5282	4.4005	–
6.8645	6.8444	4.3991	4.3812
6.8510	6.8307	4.3614	–
6.4028	6.3810	3.0738–3.7298	3.0368–3.7123
6.3994	6.1904	1.0113	0.9953
6.2118	6.1870	1.0009	0.9849
6.2084	5.9465		

**Scheme 2:**
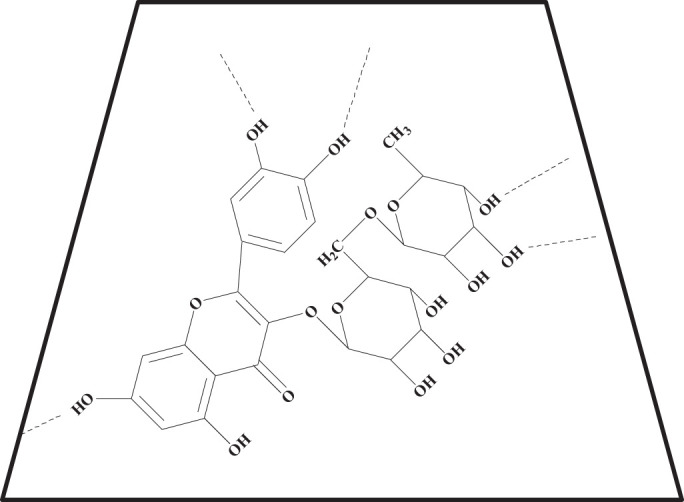
The probable structure of **R-CD** inclusion complex.

**CD** is cyclic oligosaccharides with cylindrical barrelled structures. It is reported in literature that **CD** can form inclusion complexes with **R** through the intermolecular forces of hydrogen bonding as well as hydrophobic interactions (Yan et al., 2006; Kellici et al., 2015; Savic et al., 2015). Depictions of some probable **R-CD** chemical structures can be found in Scheme 2. From these images, it can be hypothesized that molecular mobility and inter-molecular collisions between **R** molecules were reduced in frequency upon formation of the inclusion complexes. The decrease in these properties effectively prevents conveyance of energy amongst un-complexed **R** molecules. As a result of these changes, the intensity of fluorescence emission was elevated upon addition of **CD** (Zhang et al., 2011). In addition, the fluorescence emission intensity had no obvious change within 24 h (Supplementary Figure 5), which indicated that the **R-CD** was very stable.

### Ion Detection Selectivity

**FIGURE 2:**
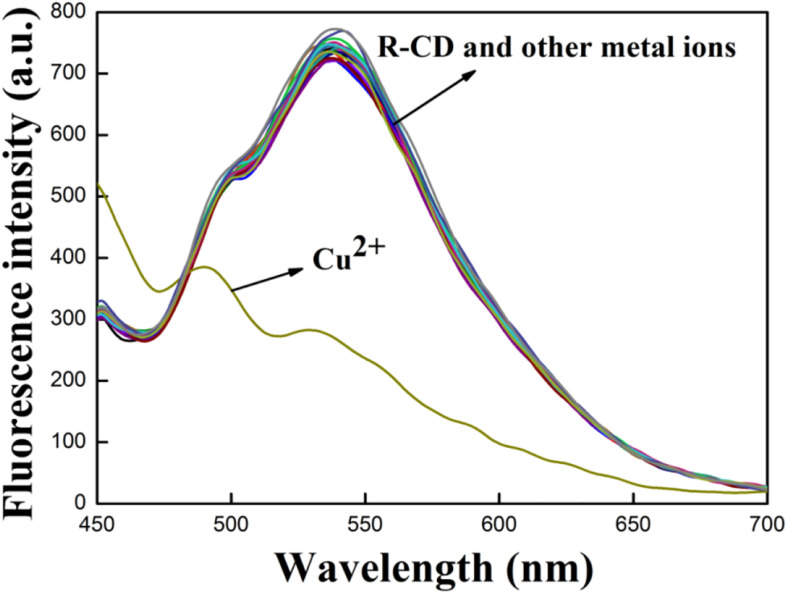
Resultant emission spectra from different cations and **R-CD** in buffer solution.

To investigate the extent of the **R-CD** inclusion complex’s selectivity toward a slection of various metal ions, a wide swath of metal cations were doped into separate **R-CD** solutions. Effects of these various cations on fluorescence intensity are shown in Figure 2. The primary finding here was that the only cation which showed any differentiation was copper ions, which provided a sharp decrease to **R-CD**’s intensity of emission. All of the other tested cations rendered no significant changes to emission spectra, even at higher concentrations. This lack of variation clearly suggested that **R-CD** could clearly recognize copper ions, and only copper ions, with good selectivity.

**FIGURE 3:**
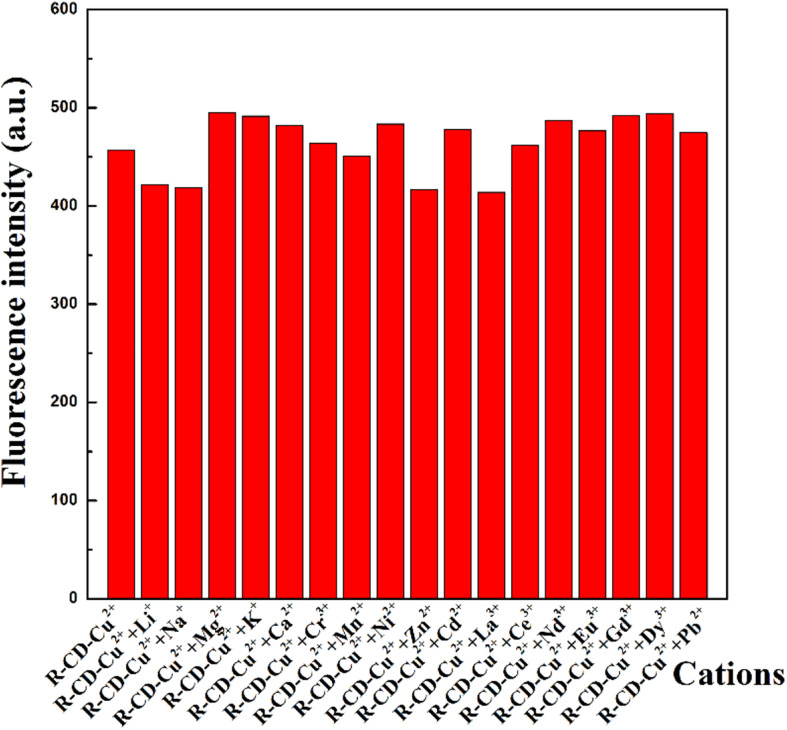
Perturbations to fluorescent intensity of **R-CD-Cu(II)** through the presence of other cations.

The next set of experiments conducted was intended to investigate how the presence of non-copper cations impact recognition of **R-CD** to copper ions. All of the previously tested cations were again analyzed through addition into a solution containing **R-CD-Cu(II)**. Resultant fluorescence spectra from these new mixtures were shown in Figure 3. From these results, it can be seen that emission intensity of **R-CD-Cu(II)** was subject to fluctuation when in the presence of the testing cations. However, these fluctuations were deemed mostly minor with respect to relative percent change, resulting in our conclusion that suggested that the recognition process of copper ions by **R-CD** retains its notable selectivity toward copper ions even when in matrices containing all of the cations tested in this work.

### Copper Detection Sensitivity With Respect to Changing [Cu^2+^]

**FIGURE 4:**
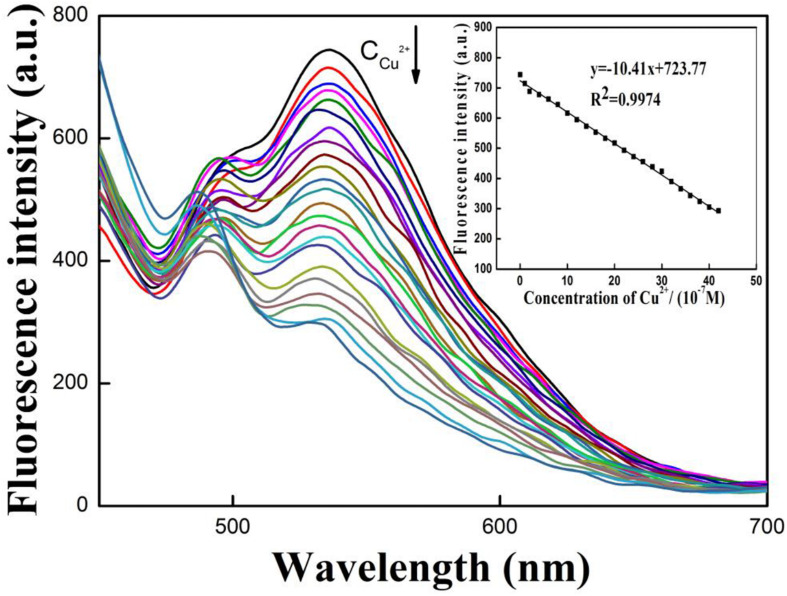
Variance in wavelength emission at increasing copper ions concentrations; quantitative regression between varying concentrations of copper ions and emission intensity in buffer solution.

From the previous results, it was next decided to quantitative define the system of **R-CD** toward detection of copper ions in solution. Results from these experiments are displayed in Figure 4. To begin, it was found that the intensity of fluorescence at 535 nm was subject to a gradual decrease when the concentration of copper ions ([Cu^2+^]) began to rise. From the intensity values over the tested range, it was found that intensity was inversely proportional to [Cu^2+^] over the concentration range of 1.0 × 10^–7^–4.2 × 10^–6^mol⋅L^–1^. Within this range, a calibration curve could be constructed. Specifically, the linear regression equation was defined as *y* = −10.41*x* + 723.77 (*R*^2^ = 0.9974), where *y* = fluorescence intensity and *x* = [Cu^2+^]. The detection limit of copper ions was 3.5 × 10^–8^ mol⋅L^–1^ (Gu et al., 2014). Based upon the relatively wide range of this regression, it can be concluded that the system is effectively sensitive to the presence of copper ions.

### Evaluating Structural Properties of the Detectable Complex

With the previous results in hand, we began to ponder the components which drive the ability to quantitatively detect copper cations with the **R-CD**. It can be speculated that the first step involved the occurrence of fluorescence quenching. In other words, copper cations engaged with **R-CD** in a way that yielded a **R-CD-Cu(II)** complex. With this final product in mind, the actual structure of the **R-CD-Cu(II)** complex was investigated via UV-visible spectrophotometry as well as Job’s plots experiments.

**FIGURE 5:**
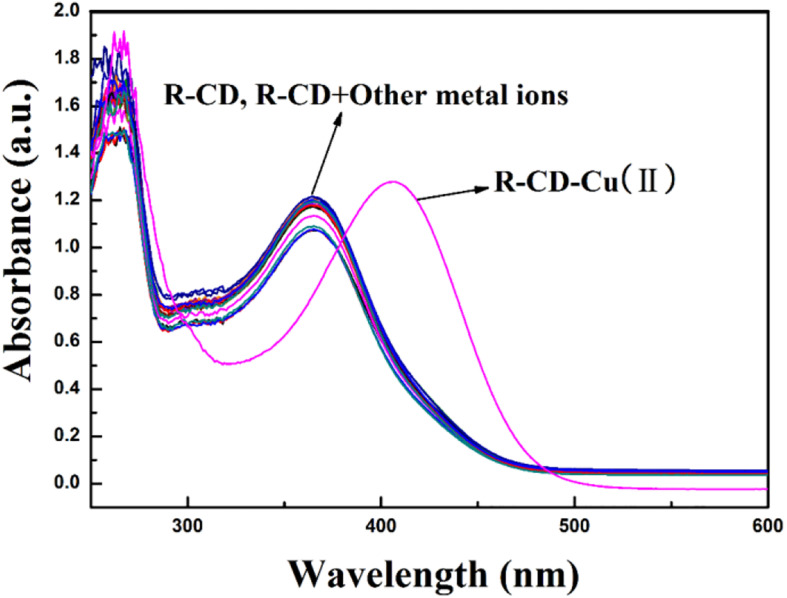
Characteristic UV-visible absorption spectra of **R-CD** in buffer solution when in the presence of copper or other tested cations.

First, the UV-visible spectra of **R-CD** and **R-CD-Cu(II)** were acquired (Figure 5). For the UV-visible spectrum of **R-CD**, it was found that there were two spectra-defining absorption bands: 375 nm (band I) and 260 nm (band II). It is important to note that both of these bands were typical for flavonoids, and they can be related back to the B-ring cinnamoyl system and the A-ring benzoyl system, respectively (Uzasc and Erim, 2014). Comparing the copper inclusion complex with neat **R-CD**, it was noted that band I shifted to 419 nm. This bathochromic shift might be best understood by the new complex effectively extending the existing conjugated system defining **R-CD**. The fact that none of the other cations demonstrated this change further demonstrates that the complexation was more specific than overall cationic charge, and was instead driven by alignment of orbitals unique to copper and **R-CD**.

**FIGURE 6:**
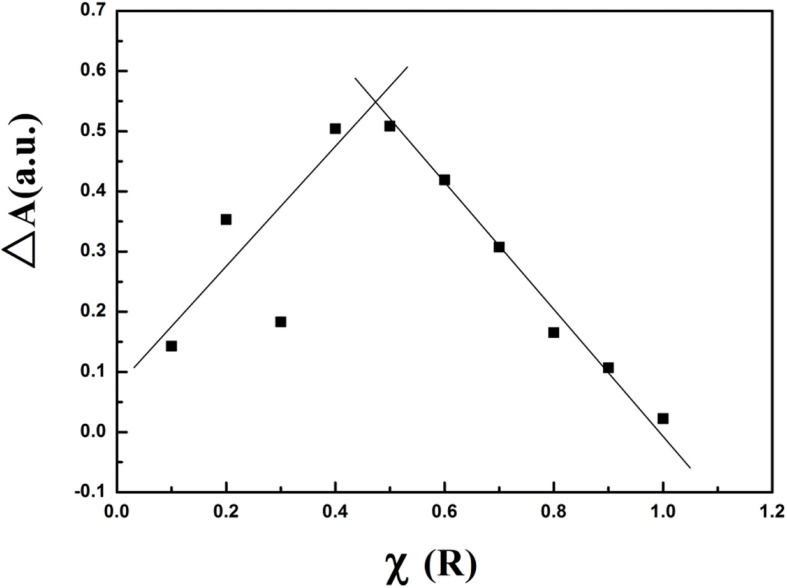
Resultant Job’s plot for **R-CD-Cu(II)** in buffer solution.

Based upon this unique configuration, we next endeavored to better understand the stoichiometry driving creation of the **R-CD-Cu(II)** complex. We decided to conduct Job’s plots experiments in order to achieve such results (Figure 6). For these experiments, changes to absorption band intensity at 419 nm was compared against molar fraction of **R** (χ) of the reactants. It was observed from the plot that maximum absorbance occurred whenχ = 0.5, which suggests that the stoichiometric ratio of **R** and copper cations was actually 1:1. Furthermore, a stability constant (K) for the resultant complex was calculated as 1.33 × 10^6^, indicating that the **R-CD-Cu(II)** complex’s stability was relatively strong and largely resistant to reversion back to the individual components. This stability is most likely driven by the stability provided to the electrons via the extension of conjugation.

**Scheme 3:**
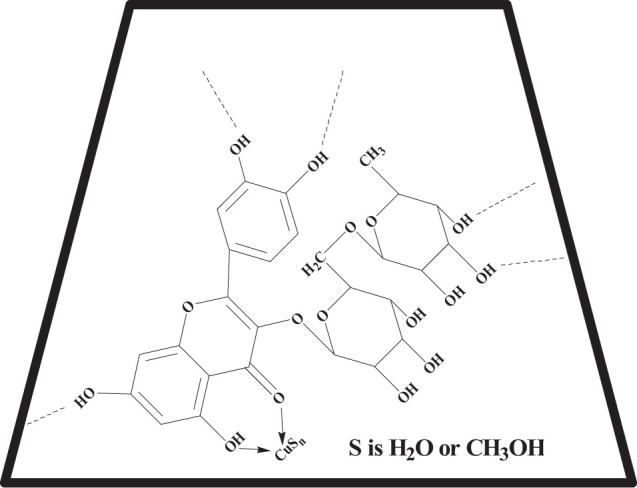
The most probable structure of **R-CD-Cu(II)** complex.

Spectra from FT-IR for **R-CD** and **R-CD-Cu(II)** are shown in Supplementary Figure 2. The key differentiator between the two spectra was a shift to 4-carbonyl. Specifically, 4-carbonyl stretching in **R-CD** was found at 1,653 cm^–1^. For the **R-CD-Cu(II)** complex, the stretching peak was shifted to 1,629 cm^–1^. This change suggests the 4-carbonyl functionality is involved in the complexation process. Based upon this clue, a hypothetical chemical structure for **R-CD-Cu(II)** complex has been included in this report as Scheme 3. In summary, it can be said that formation of **R-CD-Cu(II)** complexes is initiated by an intramolecular charge transfer driven by the energy gain from extended molecular conjugation, subsequently allowing for fluorescence quenching take place (Parveen et al., 2015).

### Determining Copper Cations Contents in Real Samples

To verify the feasibility of the quantification method described herein, we applied the approach to water samples spiked with copper. Varied amounts of copper cations were first added to pure water, and the quantification results were obtained by **R-CD** in Table 2. Compared with the content of copper cations added, the values obtained by this method were accurate. Specifically, Cu^2+^ recovery was 100.1, 99.5, and 98.8%, respectively. These promising values again demonstrated that the capability of the fluorescent sensor system developed in this work.

**TABLE 2:** Quantification of Cu^2+^ in spiked samples using **R-CD** fluorescent sensor technology.

Samples	Adding concentration (10^–7^/mol⋅L^–1^)	Measurement (10^–7^/mol⋅L^–1^)	RSD (%)	Recovery (%)
1	22	22.03 ± 0.11	0.50	100.14
2	25	24.87 ± 0.06	0.24	99.48
3	36	35.57 ± 0.10	0.28	98.81

## Conclusion

The investigated **R-CD** fluorescent sensor system was shown to selectively and accurately detect copper cations aqueous media, and was verified against spiked samples with near recovery around 100%. The mechanism for copper detection is driven by formation of the **R-CD-Cu(II)** complex, which is stabilized through an extension of the conjugated system present in **R-CD**. The resultant complex generated an ICT effect, causing fluorescence quenching. The findings of this work demonstrate that minor constituents of biomass, likely to be liberated during biorefinery processes, could be isolated in small quantities to produce materials with high-value application potential.

## Data Availability Statement

The original contributions presented in the study are included in the article/Supplementary Material, further inquiries can be directed to the corresponding author/s.

## Author Contributions

SY, LS, and ZS did the experiments and analyzed the experimental data. LX designed the research. SY and LX wrote the manuscript. All authors contributed to the article and approved the submitted version.

## Conflict of Interest

The authors declare that the research was conducted in the absence of any commercial or financial relationships that could be construed as a potential conflict of interest.
