# Supplementary material for: Extraction and Application of Natural Rutin From Sophora japonica to Prepare the Novel Fluorescent Sensor for Detection of Copper Ions
Source: Front Bioeng Biotechnol. 2021 Feb 22;9:642138. doi: 10.3389/fbioe.2021.642138 (PMC7937814; doi:10.3389/fbioe.2021.642138)
Supplement: Supplementary file 1 [file Data_Sheet_1.DOCX]

**Supplementary Material for**

**Extraction and application of natural rutin from Sophora japonica to prepare the novel fluorescent sensor for detection of copper ions**
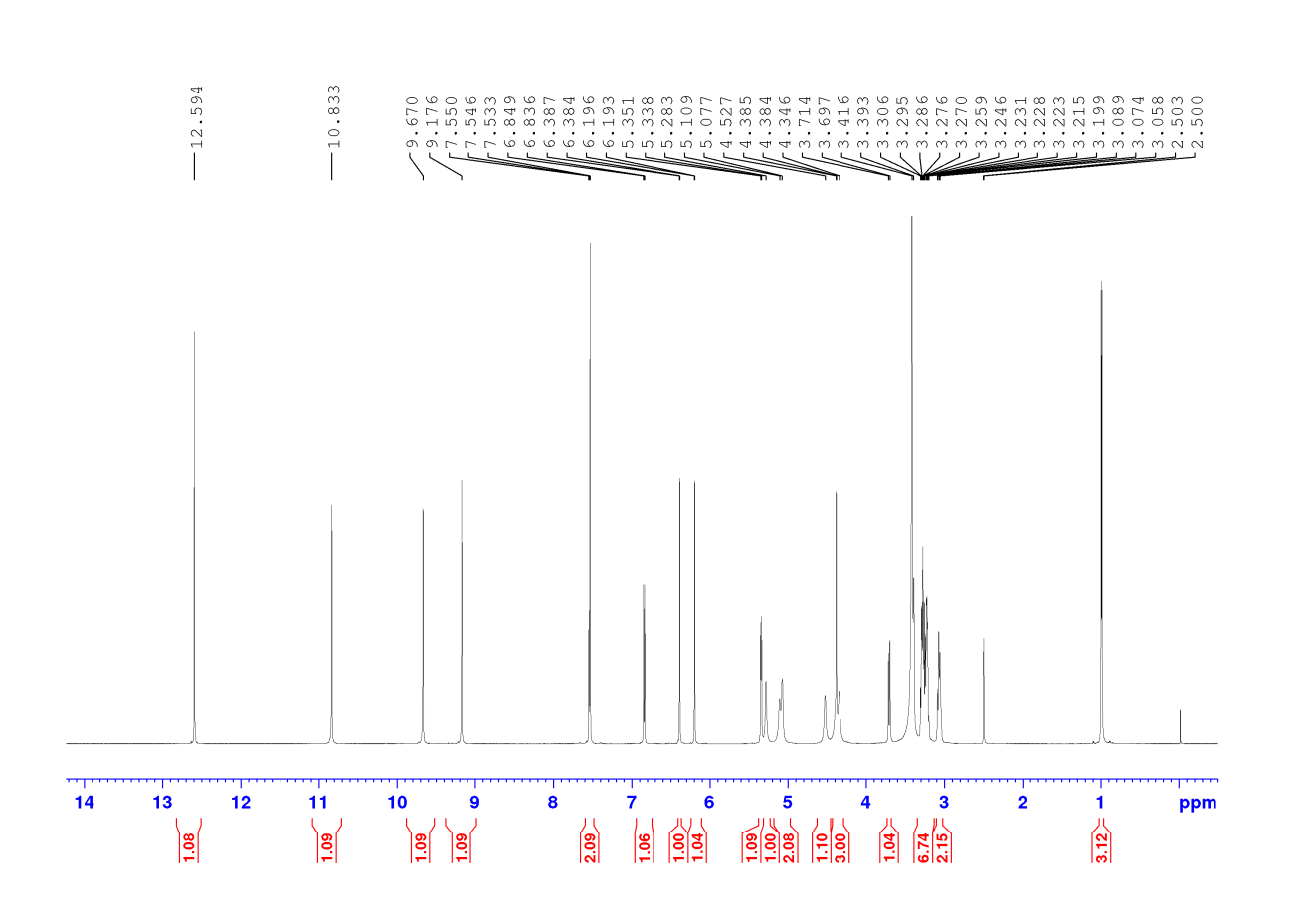


**FIGURE S1∣The ^1^HNMR spectrum of R**


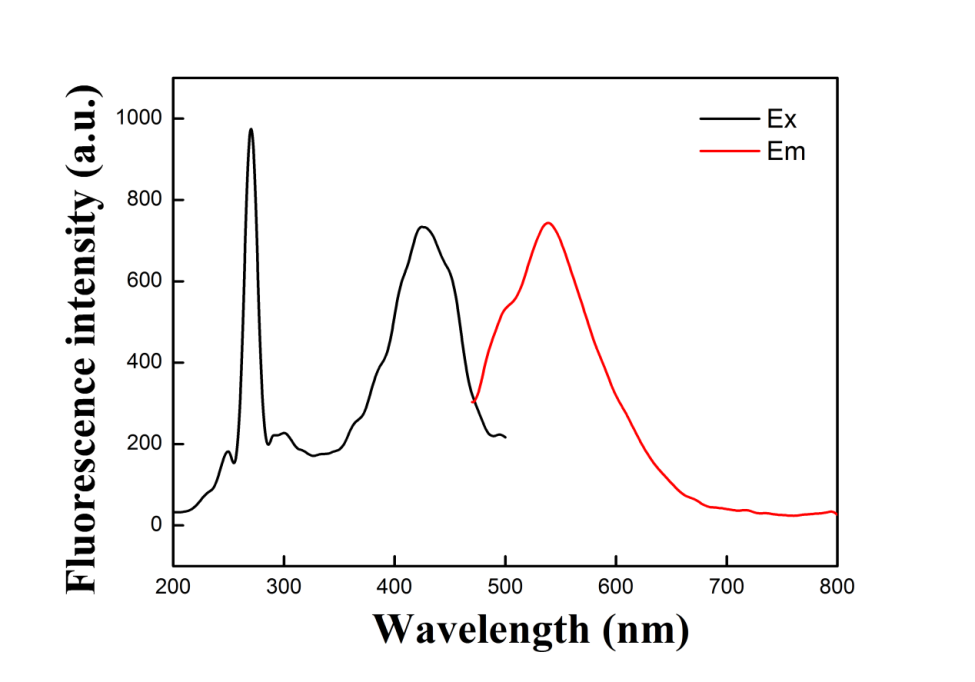


**FIGURE S2∣The excitation and emission spectra of R-CD**

The excitation (Ex) and emission (Em) spectra of **R-CD** are obtained in the figure above. There are two peaks In the Ex spectra, the peak at 268 nm is a double frequency peak induced by the instruments and samples. While the peak at 425 nm is the strongest excited wavelength, and the Em spectra is obtained with the excited wavelength at 425 nm. The maximum emission peak is at 535 nm. Stokes shift between maximum excitation and emission peak is 110 nm. At the same time, the shapes of excitation and emission peaks match each other well, thus, the best excited wavelength is 425 nm.

**
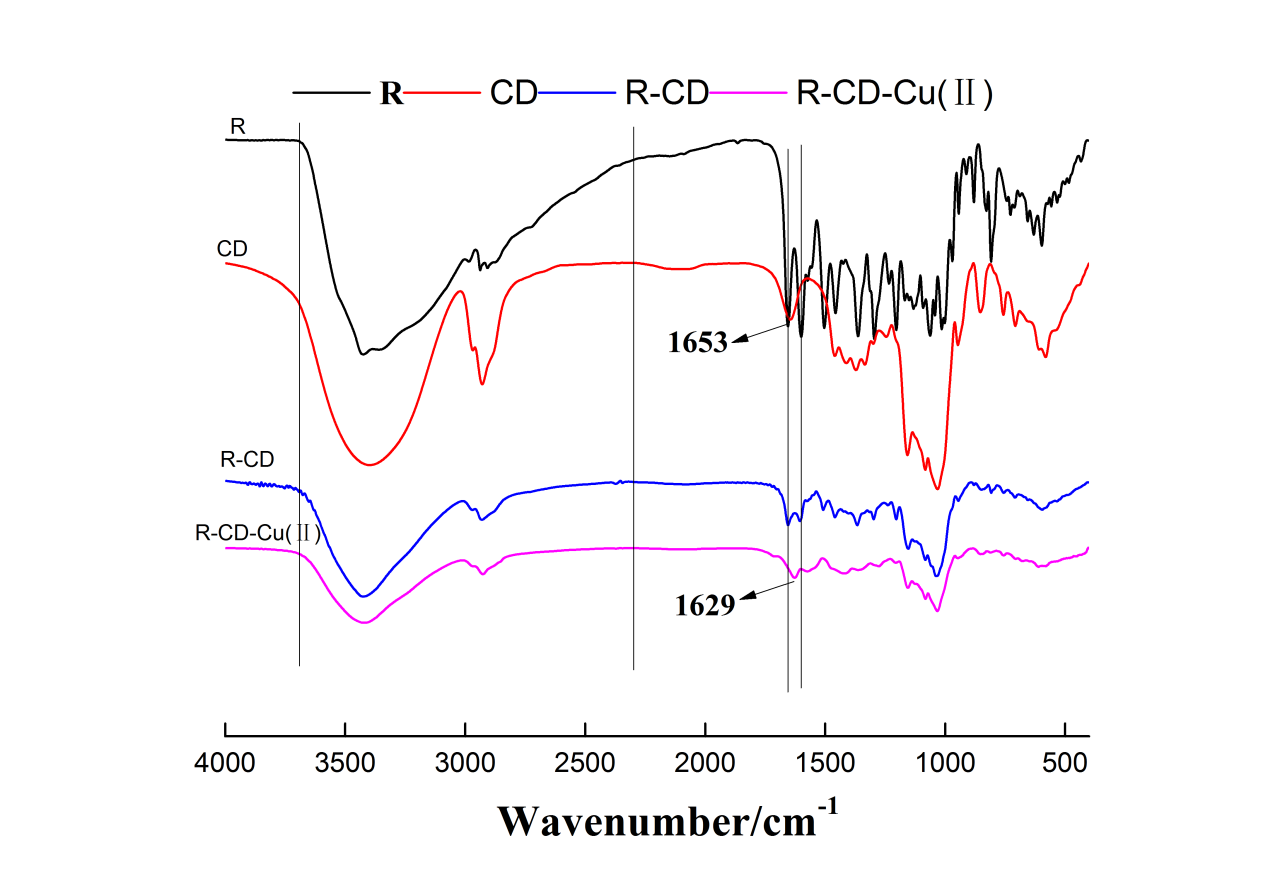
**

**FIGURE S3∣ The FT-IR spectra of R, CD, R-CD and R-CD-Cu(Ⅱ)**

**
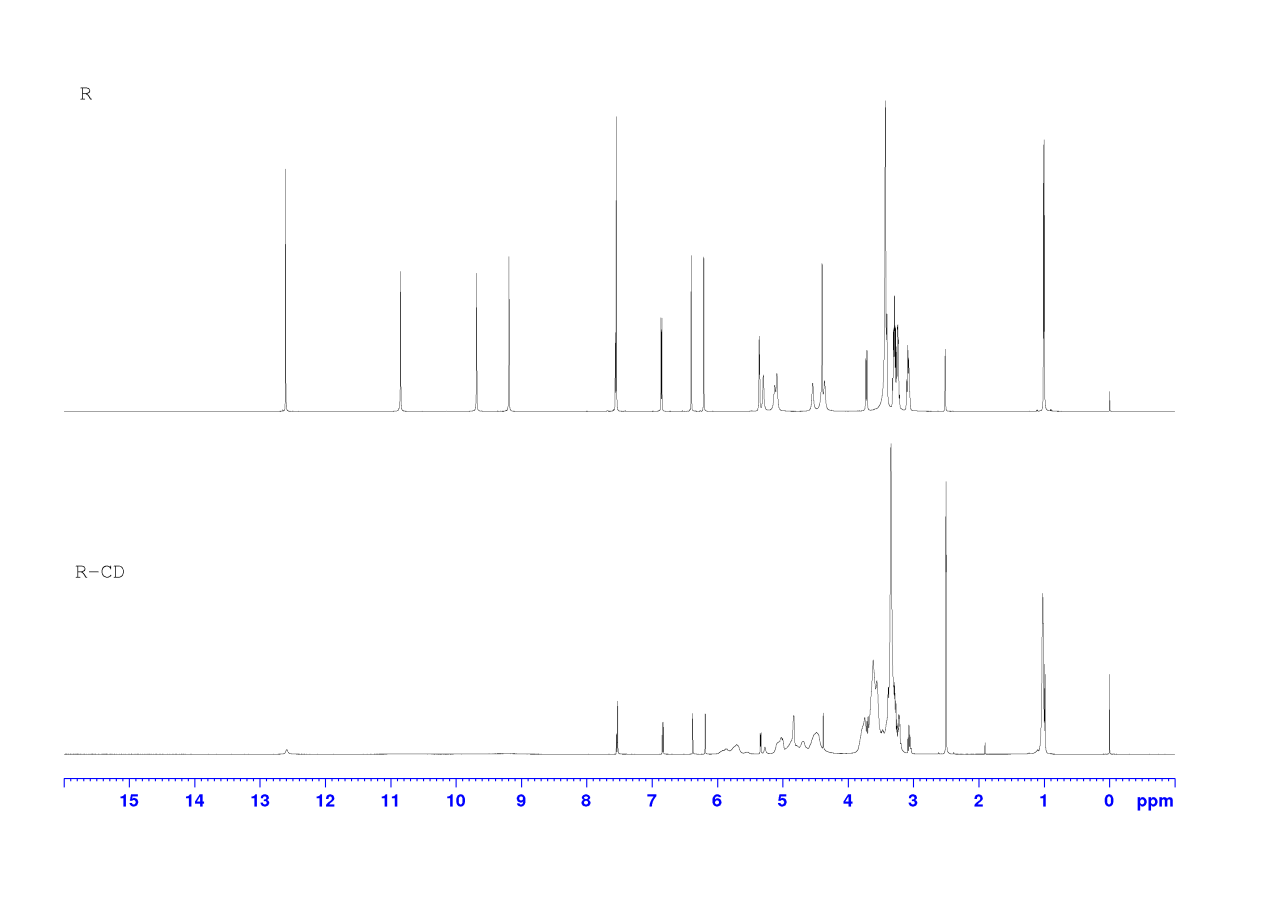
**

**(a)**

**
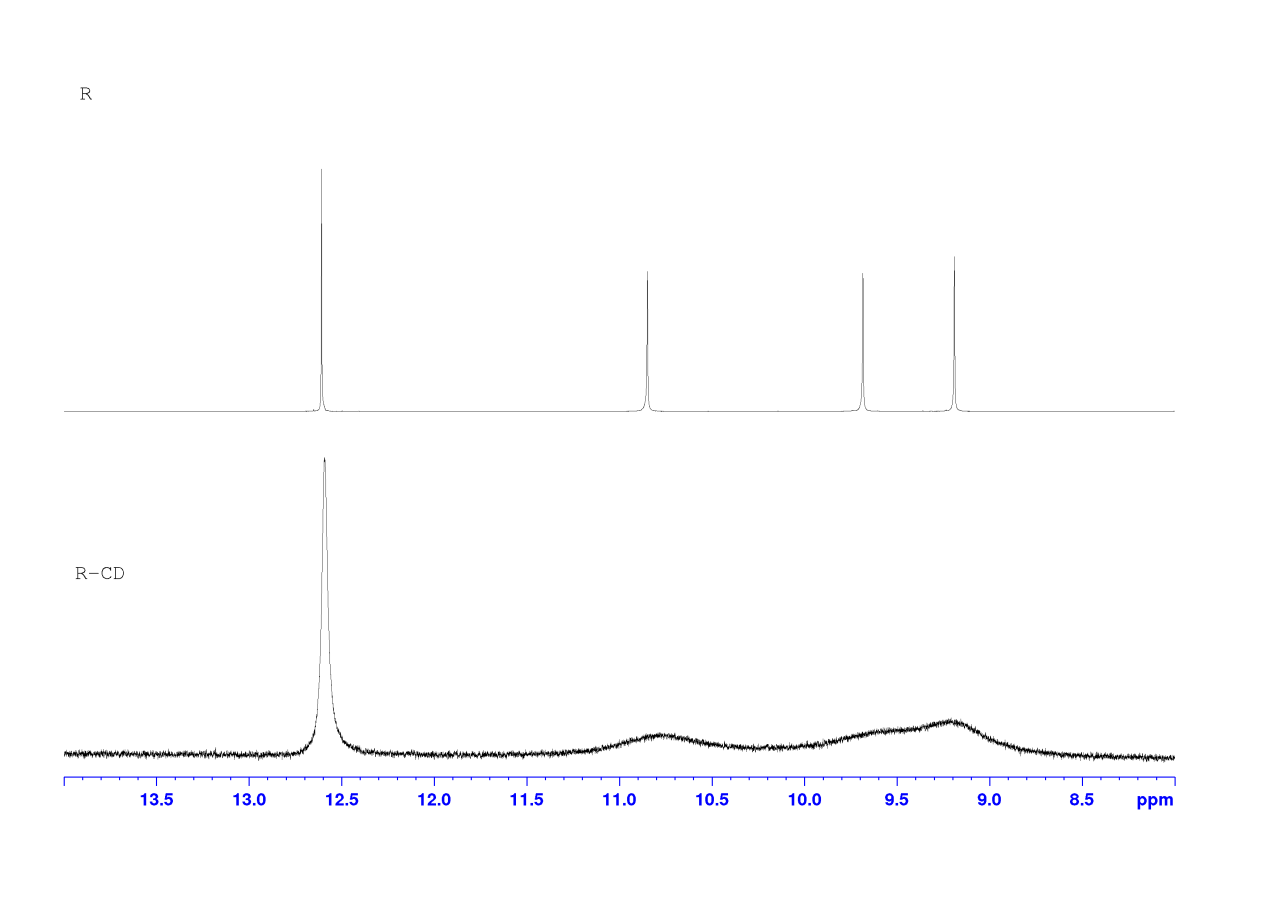
**

**(b)**

**
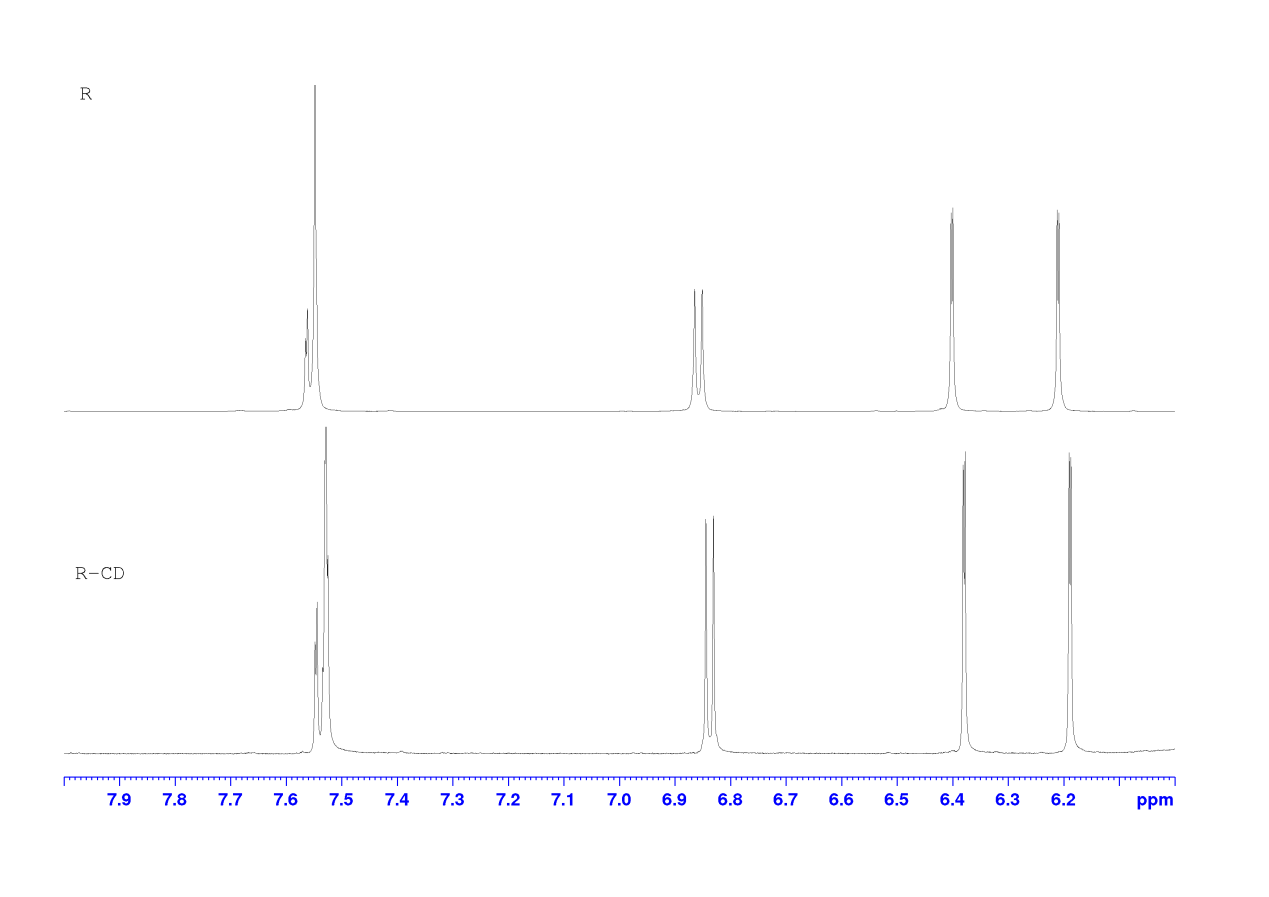
**

**(c)**

**
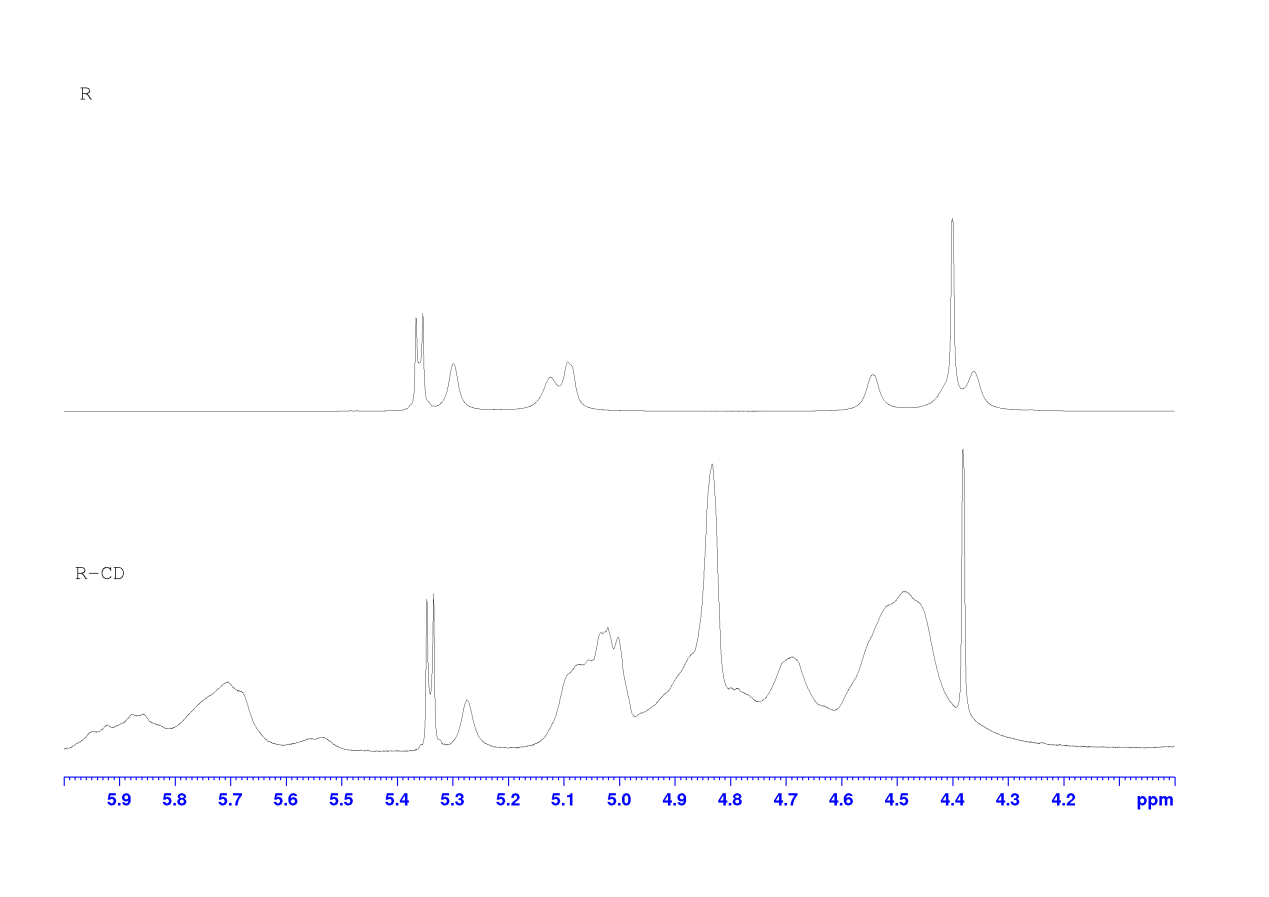
(d)**

**
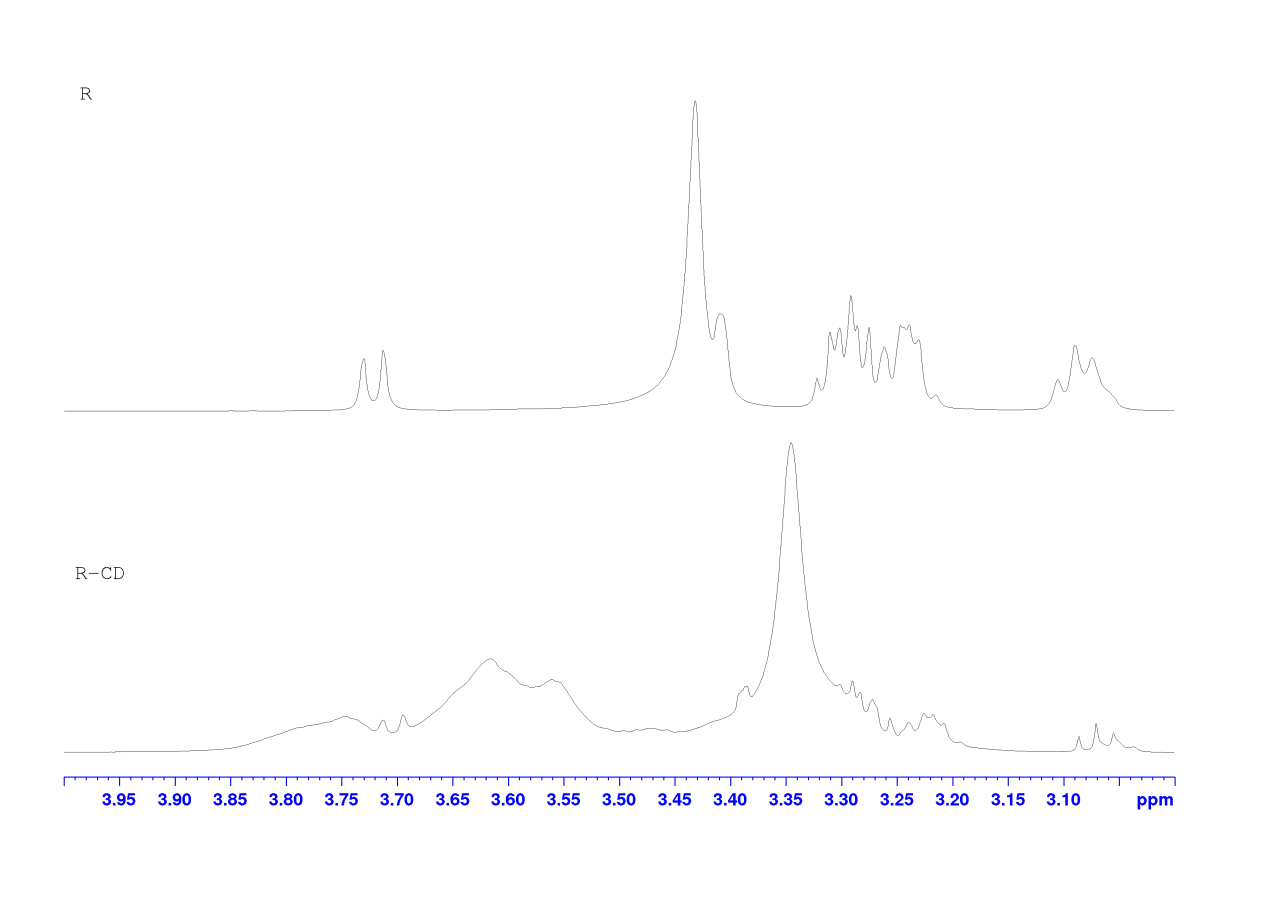
**

**(e)**

**
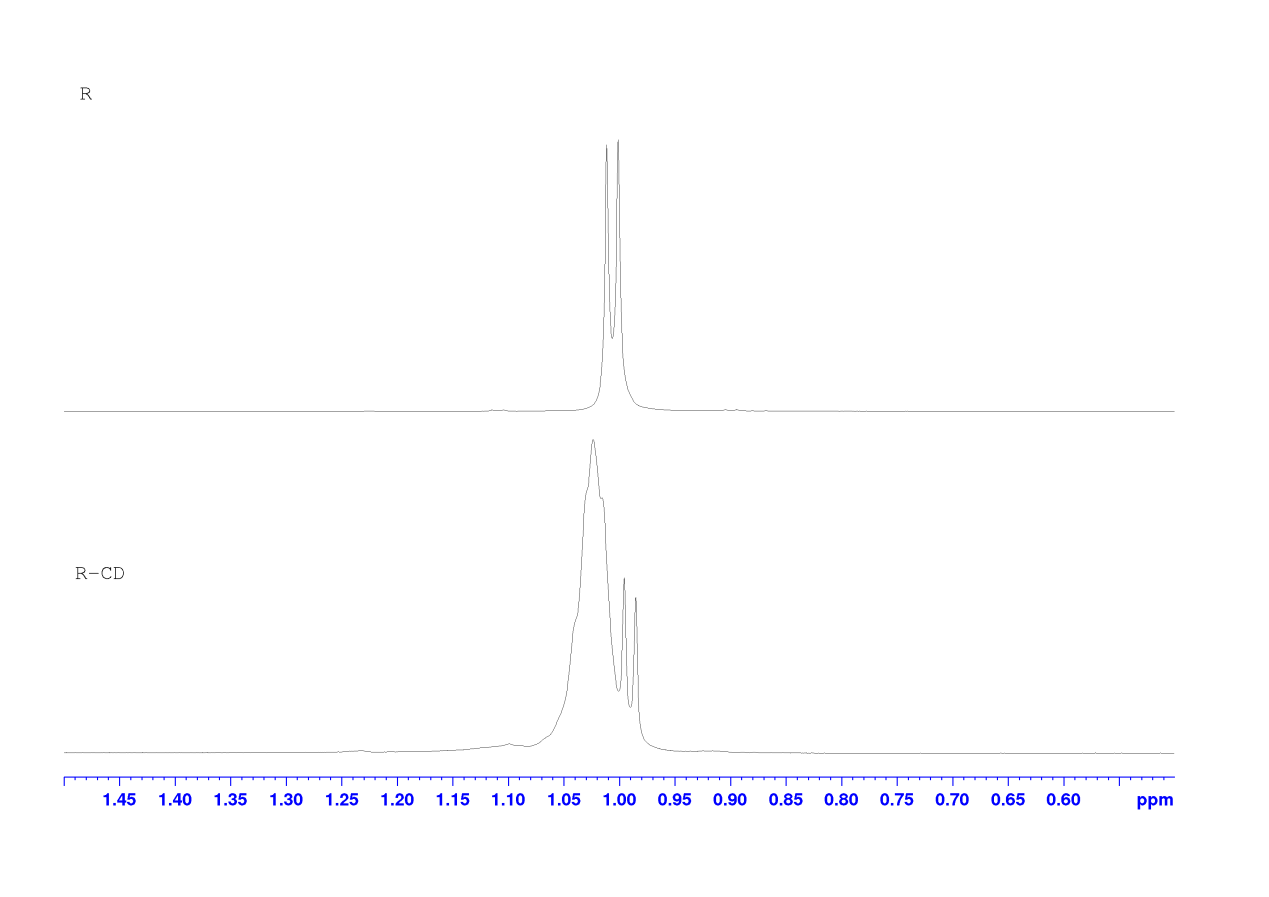
**

**(f)**

**FIGURE S4∣The ^1^HNMR spectra of R and R-CD**

**
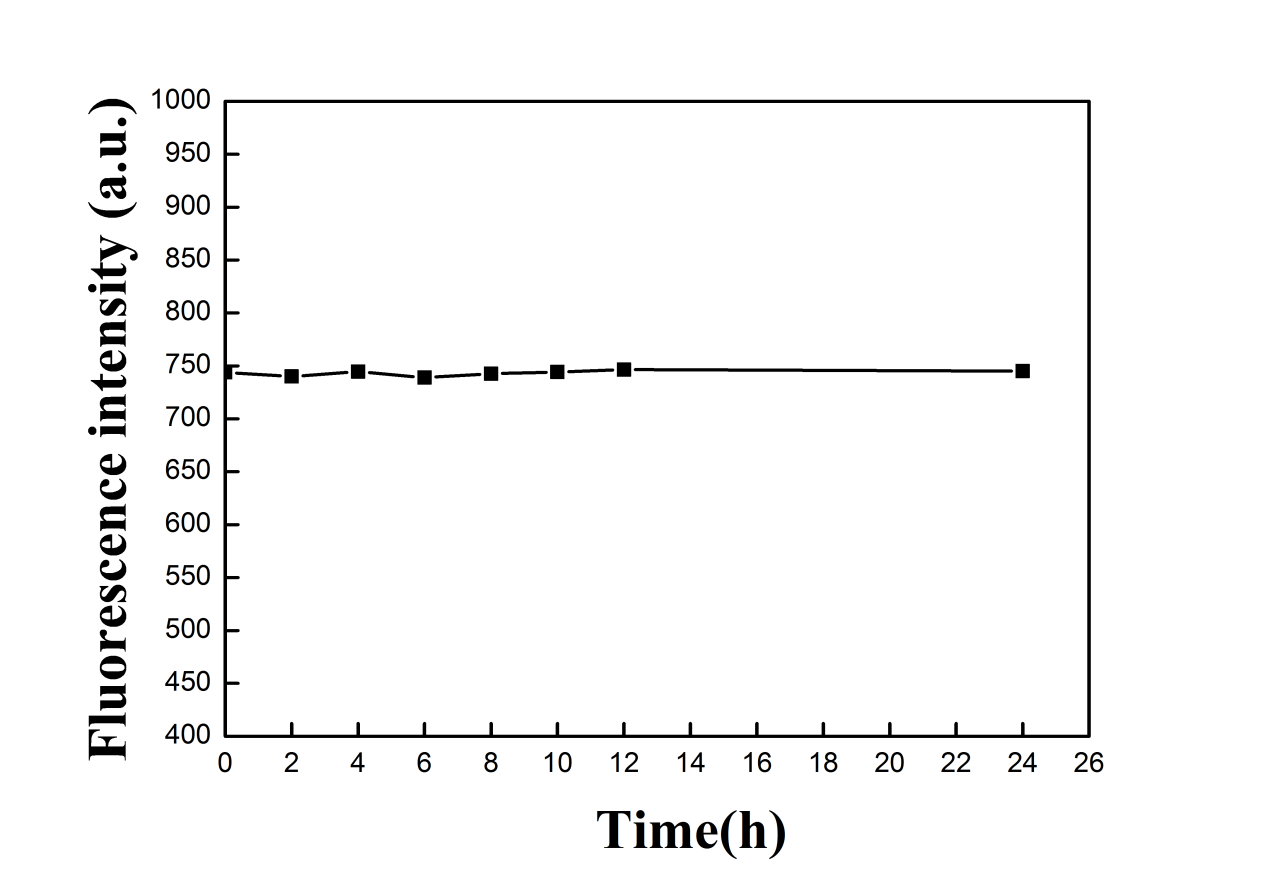
**

**FIGURE S5∣The effect of time on the fluorescence intensity of R-CD**

The fluorescence intensity of **R-CD** showed almost no change within 24 h, which means the **R-CD** is very stable.
